# Supplementary material for: Prestroke Physical Activity Matters for Functional Limitations: A Longitudinal Case–Control Study of 12,860 Participants
Source: Phys Ther. 2024 Jul 16;104(10):pzae094. doi: 10.1093/ptj/pzae094 (PMC11446638; doi:10.1093/ptj/pzae094)

SUPPLEMENTAL MATERIAL

Prestroke Physical Activity Matters for Functional Limitations: A Longitudinal Case-Control Study of 12,860 Participants

Zachary M. van Allen, Dan Orsholits, Matthieu P. Boisgontier

Table S1. People with stroke exhibiting at least slight dependency in activities of daily living (ADLs) at 1-year follow-up.

Table S2. People with stroke exhibiting at least moderate dependency in activities of daily living (ADLs) at 1-year follow-up.

Table S3. People with stroke exhibiting severe or total dependency in activities of daily living (ADLs) at 1-year follow-up.

Table S4. People with stroke who are moderately active in instrumental activities of daily living (IADLs) at 1-year follow-up.

Table S5. People with stroke who are inactive in instrumental activities of daily living (IADLs) at 1-year follow-up.

Table S6. Activities of daily living (ADLs) and instrumental activities of daily living (IADLs) counts for people with stroke and adults without stroke. Counts reflect the number of I/ADL limitations reported.

Table S7. Frequency of moderate and vigorous physical activity in people with stroke and adults without stroke at baseline.

Table S8. Counts of physically active and inactive participants in people with stroke and adults without stroke.

Table S9. Results of the sensitivity analyses testing the interaction between stroke-related status and physical activity (“hardly ever or never” vs. “at least once a month”) on limitations in activities of daily living (ADLs) and instrumental activities of daily living (IADLs).

Figure S1. Result of the sensitivity analysis showing the effect of physical activity (“Hardly ever or never” vs. “At least once a month”) on limitations in activities of daily living (ADLs) and instrumental activities of daily living (IADLs) in people with stroke and matched adults without stroke over time.

Table S1. People with stroke exhibiting at least slight dependency in activities of daily living (ADLs) at 1-year follow-up

Study	Outcome Measure	Threshold	**Sample Size** (n)	**Dependent Survivors** (%)
Appelros (2007)	Barthel Index	<20/20	246	39.0
Ayerbe (2011)	Barthel Index	<20/20	1732	67.0
Carolei (1997)	Barthel Index	<20/20	517	61.7
Dhamoon (2009)	Barthel Index	<95/100	525	48.1
Gil-Salcedo (2022)	Modified Ranking Scale	>1/6	3718	63.8
Hartman-Maeir (2007)	FIM motor scale	<91/91	56	68.0
Leśniak (2008)	Barthel Index	<20/20	80	43.7
Mar (2015)	Barthel Index	<100/100	250	47.2
Minelli (2007)	Barthel Index	<100/100	79	57.0
Skånér (2007)	Katz ADL	<6/6	135	31.9
Sveen (1996)	Barthel Index	<20/20	74	58.1
Taub (1994)	Barthel Index	<20/20	225	34.0
van de Port (2006)	Barthel Index	<19/20	264	40.1
Willey (2010)	Barthel Index	<95/100	246	44.7
Wong (2014)	Modified Ranking Scale	>1/6	194	64.4
**Total n**			**8341**	
**Weighted mean (%)**				**59.2**

Note. FIM = Functional Independent Measure.

Table S2. People with stroke exhibiting at least moderate dependency in activities of daily living (ADLs) at 1-year follow-up.

**Study**	**Outcome Measure**	**Threshold**	**Sample Size** (n)	**Dependent Survivors (%)**
Appelros (2007)	Barthel Index	<15/20	246	31.2
Broussy (2019)	Modified Ranking Scale	>2/6	161	29.6
De Campos (2017)	Modified Ranking Scale	>2/6	287	16.4
Jokinen (2015)	Modified Ranking Scale	>2/6	364	44.0
López-Cancio (2017)	Modified Ranking Scale	>2/6	143	53.8
Mar (2015)	Barthel Index	<90/100	250	40.4
Patel (2002)	Barthel Index	<15/20	619	36.2
Patel (2003)	Barthel Index	<15/20	136	36.0
Santus (1990)	Barthel Index	<75/100	76	46.1
Taub (1994)	Barthel Index	<15/20	225	11.0
Urbanek (2018)	Modified Ranking Scale	>2/6	1119	41.6
Verhoeven (2011)	Barthel Index	<18/20	92	38.0
Wafa (2020)	Barthel Index	<15/20	1961	24.1
Wolfe (2011)	Barthel Index	<15/20	1578	13.1
Wong (2014)	Modified Ranking Scale	>2/6	194	33.0
**Total** (n)			**7451**	
**Weighted mean** (%)				**32.9**

Table S3. People with stroke exhibiting severe or total dependency in activities of daily living (ADLs) at 1-year follow-up

**Study**	**Outcome Measure**	**Threshold**	**Sample Size** (n)	**Dependent Survivors** (%)
Appelros (2007)	Barthel Index	<12/20	246	16.0
Broussy (2019)	Barthel Index	<12/20	161	12.7
Dhamoon (2009)	Barthel Index	<60/100	525	18.0
Gil-Salcedo (2022)	Modified Ranking Scale	>3/6	3718	27.3
Mar (2015)	Barthel Index	<60/100	250	20.4
Patel (2002)	Barthel Index	<10/20	619	9.4
Patel (2003)	Barthel Index	<10/20	136	15.4
Willey (2010)	Barthel Index	<60/100	246	15.9
Wong (2014)	Modified Ranking Scale	>3/6	194	19.6
**Total n**			**6095**	
**Weighted mean (%)**				**22.6**

Table S4. People with stroke who are moderately active in instrumental activities of daily living (IADLs) at 1-year follow-up.

**Study**	**Outcome Measure**	**Threshold**	**Sample Size** (n)	**Dependent Survivors** (%)
Appelros (2007)	Frenchay Activities Index	<30/45	246	78.8
Ayerbe (2011)	Frenchay Activities Index	<30/45	1403	79.7
Patel (2002)	Frenchay Activities Index	<30/45	619	85.7
Patel (2003)	Frenchay Activities Index	<30/45	136	88.2
Sveen (1996)	Frenchay Activities Index	<29/45	74	75.6
**Total n**			**2478**	
**Weighted mean (%)**				**81.5**

Table S5. People with stroke who are inactive in instrumental activities of daily living (IADLs) at 1-year follow-up.

Study	Outcome Measure	Threshold	Sample Size (n)	Dependent Survivors (%)
Appelros (2007)	Frenchay Activities Index	<15/45	246	46.3
Patel (2002)	Frenchay Activities Index	<15/45	619	40.4
Patel (2003)	Frenchay Activities Index	<15/45	136	72.7
van de Port (2006)	Frenchay Activities Index	<15/45	264	35.2
Wolfe (2011)	Frenchay Activities Index	<15/45	1578	38.8
**Total n**			**2843**	
**Weighted mean (%)**				**41.1**

Table S6. Activities of daily living (ADLs) and instrumental activities of daily living (IADLs) counts for people with stroke and adults without stroke. Counts reflect the number of I/ADL limitations reported.

Activities of Daily Living (ADLs)								
**People With Stroke**								
**ADL**	**Wave 1**	**Wave 2**	**Wave 3**	**Wave 4**	**Wave 5**	**Wave 6**	**Wave 7**	**Wave 8**
0	929	1162	NA	1287	1389	1307	1115	795
1	68	92	NA	151	166	187	188	139
2	20	36	NA	62	89	125	126	70
3	11	23	NA	48	45	58	59	57
4	4	10	NA	22	33	40	53	36
5	2	7	NA	31	29	50	51	45
6	2	11	NA	34	57	72	86	79
NA	1125	820	2161	526	353	322	483	940
**Adults Without Stroke**								
**ADL**	**Wave 1**	**Wave 2**	**Wave 3**	**Wave 4**	**Wave 5**	**Wave 6**	**Wave 7**	**Wave 8**
0	4627	5964	NA	7258	7770	7685	7077	4974
1	259	391	NA	624	636	734	694	491
2	91	130	NA	210	257	316	289	207
3	36	59	NA	117	141	130	150	125
4	29	40	NA	70	94	111	81	82
5	3	29	NA	53	79	84	102	55
6	16	21	NA	71	78	95	130	105
NA	5744	4171	10805	2402	1750	1650	2282	4766

Instrumental Activities of Daily Living (ADLs)								
**People With Stroke**								
**IADL**	**Wave 1**	**Wave 2**	**Wave 3**	**Wave 4**	**Wave 4**	**Wave 6**	**Wave 7**	**Wave 8**
0	869	1050	NA	1158	1201	1091	931	625
1	104	154	NA	203	233	272	216	154
2	31	63	NA	103	113	130	140	105
3	14	23	NA	48	66	93	100	67
4	9	13	NA	26	58	67	73	68
5	6	10	NA	29	39	57	60	52
6	1	15	NA	23	31	49	57	41
7	2	13	NA	45	67	80	101	109
NA	1125	820	2161	526	353	322	483	940
**Adults Without Stroke**								
**IADL**	**Wave 1**	**Wave 2**	**Wave 3**	**Wave 4**	**Wave 5**	**Wave 6**	**Wave 7**	**Wave 8**
0	4315	5490	NA	6726	7109	6968	6291	4371
1	458	648	NA	857	912	1016	945	720
2	157	239	NA	351	387	444	437	289
3	63	108	NA	153	205	248	247	193
4	30	64	NA	104	150	180	190	139
5	14	38	NA	71	95	97	160	104
6	10	23	NA	48	73	74	97	77
7	14	24	NA	93	124	128	156	146
NA	5744	4171	10805	2402	1750	1650	2282	4766

Table S7. Frequency of moderate and vigorous physical activity in people with stroke and adults without stroke at baseline.

Physical Activity Frequency	**People With Stroke**		**Adults Without Stroke**	
	Moderate Physical Activity	Vigorous Physical Activity	Moderate Physical Activity	Vigorous Physical Activity
Hardly ever or never	258	931	1086	4291
One to three times a month	127	216	575	1050
Once a week	274	306	1415	1501
More than once a week	1500	706	7702	3937
NA	2	2	27	26

Notes. The question for assessing moderate physical activity was "How often do you engage in activities that require a low or moderate level of energy such as gardening, cleaning the car, or doing a walk?". The question for assessing vigorous physical activity was "How often do you engage in vigorous physical activity, such as sports, heavy housework, or a job that involves physical labor?

Table S8. Counts of physically active and inactive participants in people with stroke and adults without stroke.

Type of Analysis	**People With Stroke**		**Adults Without Stroke**	
	Physically Inactive	Physically Active	Physically Inactive	Physically Active
Main Analyses	595	1564	2720	8060
Sensitivity Analyses	1202	957	6343	4434

Notes. In the main analyses, participants who answered "more than once a week" to at least one of the questions were classified as physically active, whereas the other participants were classified as physically inactive. In the sensitivity analyses, participants who answered "hardly ever or never" to one of the two questions related to the level of physical activity were classified as physically inactive, whereas the other participants were classified as physically active.

Table S9. Results of the sensitivity analyses testing the interaction between stroke-related status and physical activity (hardly ever or never vs. at least once a month) on limitations in activities of daily living (ADLs) and instrumental activities of daily living (IADLs).

	ADLs		IADLs	
**Exposures**	**b (95 CI)**	***p***	**b (95 CI)**	***p***
Intercept	-0.563 (-0.653 to -0.474)	$< 2.0 \times 10^{-16}$	-1.137 (-1.255 to -1.019)	$< 2.0 \times 10^{-16}$
Stroke	-0.044 (-0.089 to 0.001)	0.056	-0.045 (-0.100 to 0.010)	0.107
Physical Activity	0.223 (0.198 to 0.247)	$< 2.0 \times 10^{-16}$	0.317 (0.285 to 0.348)	$< 2.0 \times 10^{-16}$
Wave	0.016 (0.005 to 0.027)	0.003	0.017 (0.003 to 0.030)	0.015
$Wave^2$	0.006 (0.005 to 0.008)	1.1×10^{-14}	0.013 (0.011 to 0.015)	$< 2.0 \times 10^{-16}$
Age	0.007 (0.005 to 0.008)	$< 2.0 \times 10^{-16}$	0.012 (0.011 to 0.014)	$< 2.0 \times 10^{-16}$
Sex	0.063 (0.041 to 0.085)	3.0×10^{-08}	0.192 (0.164 to 0.221)	$< 2.0 \times 10^{-16}$
Education				
Primary (vs. Secondary)	0.117 (0.091 to 0.142)	$< 2.0 \times 10^{-16}$	0.224 (0.191 to 0.256)	$< 2.0 \times 10^{-16}$
Tertiary (vs. Secondary)	-0.026 (-0.055 to 0.003)	0.074	-0.047 (-0.083 to -0.011)	0.011
Chronic Conditions	0.113 (0.099 to 0.128)	$< 2.0 \times 10^{-16}$	0.178 (0.160 to 0.196)	$< 2.0 \times 10^{-16}$
Stroke × Physical Activity	0.084 (0.026 to 0.142)	0.004	0.062 (-0.012 to 0.136)	0.098
Stroke × Wave	0.022 (-0.005 to 0.048)	0.109	0.032 (0.000 to 0.065)	0.052
Stroke × $Wave^2$	0.014 (0.010 to 0.018)	7.9×10^{-13}	0.020 (0.015 to 0.025)	6.7×10^{-15}

Notes. 95 CI = 95% confidence interval, ADLs = activities of daily living, IADLs = instrumental activities of daily living

Figure S1. Result of the sensitivity analysis showing the effect of physical activity (PA; "Hardly ever or never" vs. "At least once a month") on limitations in activities of daily living (ADLs) and instrumental activities of daily living (IADLs) in people with stroke and matched adults without stroke over time.

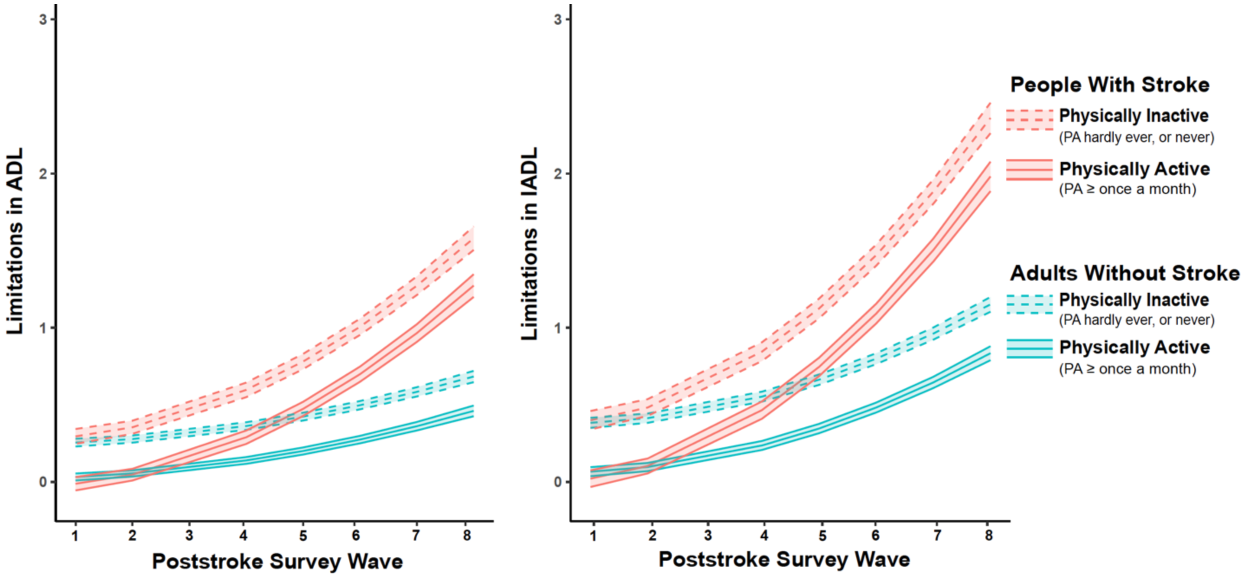

Supplement: 2023-0704_R1_2024-07-11_SUPPLEMENTAL_MATERIAL_pzae094 [file 2023-0704_r1_2024-07-11_supplemental_material_pzae094.pdf]
